# Generative artificial intelligence tools in journal article preparation: A preliminary catalog of ethical considerations, opportunities, and pitfalls*

**DOI:** 10.3168/jdsc.2024-0707

**Published:** 2025-01-06

**Authors:** Robin R. White

**Affiliations:** School of Animal Sciences, Virginia Tech, Blacksburg, VA 24060

## Abstract

The launch of generative artificial intelligence (GenAI) tools has catalyzed considerable discussion about the potential impacts of these systems within the scientific article preparation process. This symposium paper seeks to summarize current recommendations on the use of GenAI tools in scientific article preparation, and to provide speculations about the future challenges and opportunities of GenAI use in scientific publishing. Due to the dynamic nature of these tools and the rapid advancement of their sophistication, the most important recommendation is that ongoing engagement and discussion within the scientific community about these issues is critical. When using GenAI tools in scientific article preparation, humans are ultimately accountable and responsible for products produced. Given that accountability, an expert panel convened by the National Academies of Science, Engineering, and Medicine recently proposed principles of GenAI use in science communication, including (1) transparent disclosure and attribution; (2) verification of AI-generated content and analyses; (3) documentation of artificial intelligence (AI)-generated data; (4) a focus on ethics and equity; and (5) continuous monitoring, oversight, and public engagement. In addition to the importance of human accountability, many publishers have established consistent policies suggesting that GenAI tools should not be used for peer reviewing, figure generation or manipulation, or assigned authorship on scientific articles. Along with the potential ethical challenges associated with GenAI use in scientific publishing, there are numerous potential benefits. Herein we summarize example conversations demonstrating the capacity of GenAI tools to support the article preparation process, and an example standard operating procedure for human-AI interaction in article preparation. Finally, diverse broader questions about the impact of GenAI tools on communication, knowledge, and advancement of science are raised for rumination.

## Highlights

• Dairy foods are available in a variety of physical forms (i.e., liquid, semi-solids, and powders).
• Changing formulation or processing conditions is an effective way of controlling functionality.
• Material science approaches help to understand the structure-function relationships in foods.

## Graphical Abstract

**Figure:**
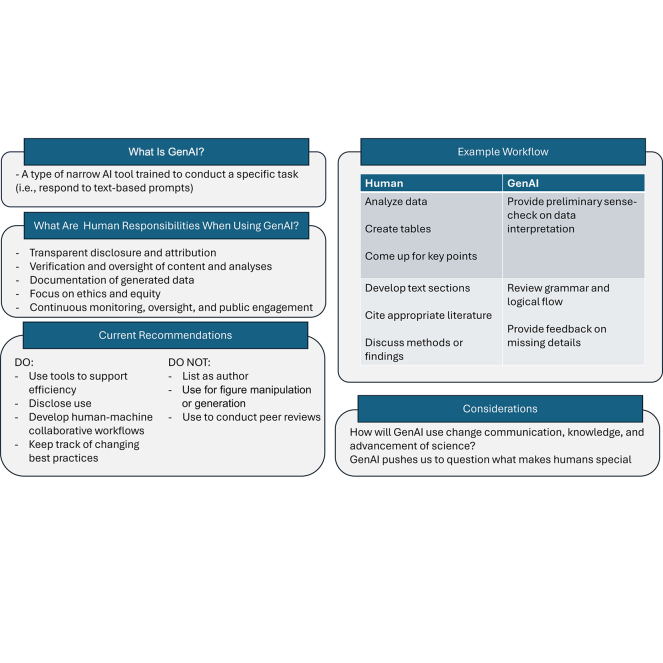
**Summary:** Understanding the structure-function relationship in various dairy food matrices is important for improving the profitability and efficiency of commercial dairy manufacturing operations. Composition and processing conditions of food matrices determine the functionality of dairy foods. Based on the desired functionality of the food, material properties can be used to understand the arrangement of structure at various length scales. Precise measurement of food structure and material properties is important in optimizing functionality through optimum design of equipment or processes. In this work, we have tried to show that the use of novel material science approaches can not only be helpful in understanding the structure-function relationship in various dairy food matrices (slicing of cheese, powder rheology, high-solid spray drying, and cold gelling behavior of highly concentrated micellar casein concentrate) but also in potentially improving the profitability and efficiency of commercial dairy manufacturing operations.

Since the preliminary launch of publicly accessible generative artificial intelligence (**GenAI**) tools, the scientific literature has expanded to include considerable discourse about the role of these tools in preparation of scientific articles, and in science communication and data sharing more broadly (Conroy, 2023; Bagenal, 2024; Chauhan and Currie, 2024). Early literature ranged considerably in advocacy, with some sources suggesting GenAI will be critical to the future of science communication and data analysis, and others advising caution in the use of GenAI to support journal article preparation (Lund et al., 2023; Noy and Zhang, 2023; Salvagno et al., 2023). Gradually, a degree of consensus has emerged reflecting cautious and judicious use of GenAI tools to support, but not replace, the role of humans in journal article preparation (Blau et al., 2024; Ganjavi et al., 2024).

The goals of this symposium article are to summarize recommended practices on use of GenAI in journal article preparation, and to provide one author's speculation and questions on future directions for GenAI use in science communication and data sharing more broadly. Undoubtedly, as we gain greater familiarity with this technology, the relevance of these recommendations will shift. Therefore, the need for regular and ongoing discourse on the subject is critical to ensure best practices are identified, followed, and improved over time.

An expert committee commissioned by the National Academies of Science, Engineering, and Medicine proposed 5 principles of human accountability and responsibility when using GenAI tools in science communication (Blau et al., 2024). These principles include (1) transparent disclosure and attribution; (2) verification of GenAI-produced content and analyses; (3) documentation of AI-generated data; (4) a focus on ethics and equity; and (5) continuous monitoring, oversight, and public engagement (Blau et al., 2024). These principles co-evolved with important cautionary recommendations that have been adopted by many journals. For example, toward principle 1, many journals require specific disclosure of how GenAI tools were used in article preparation (Ganjavi et al., 2024). This recommendation evolved in lieu of using GenAI as an author, because GenAI tools are classified as narrow artificial intelligence (**AI**; Singh et al., 2024), are trained to conduct a single task, and do not meet the standards for authorship for most journals (Lund and Naheem, 2024). Toward principle 2, the use of GenAI tools to support peer reviewing of papers or grant proposals is viewed as a potential breach of confidentiality and is recommended against. This is because GenAI tools are typically trained through reinforcement learning, allowing for continuous ingesting of data to refine the model (Sutton and Barto, 1998). As such, many existing GenAI tools retain user input and exchanges as a part of their (ever growing) training data, and it is recommended that authors be careful about the kind of information they input into GenAI tools due to the potential ethical challenges with unauthorized sharing of privileged data and ideas (Kim, 2024; Mollaki, 2024; Nath et al., 2024). Principle 3 focuses on the use of GenAI to synthesize data, which is a topic meriting its own review due to the diverse issues associated with using models to produce data. Toward principle 4, GenAI is not recommended for scientific figure preparation by many journals (Gu et al., 2022), due in part to the historical challenges associated with image manipulation within scholarly publications (Cromey, 2010), and because GenAI figures lack reproducibility and traceability (Al-kfairy et al., 2024). Finally, toward principle 5, there is considerable dialog highlighting the need for ongoing discussion about acceptable use of GenAI led by many top-ranked publishers and journals. Although not all journals have consistent policies, these boundaries represent the most up-to-date recommendation from the majority of publishers of scientific literature (Ganjavi et al., 2024). As GenAI systems develop, particularly for specialized functions related to science communication and journal article preparation, these guidelines will adapt accordingly.

Although the rapidly shifting environment around GenAI tools makes a comprehensive review of contemporary tools impractical, there is value in highlighting some example systems and how they can contribute to journal article preparation. Writing is a skill that requires practice and feedback, copyediting and content generation, and creativity to help avoid writer's block (Grogan, 2021), among other things. Generalist tools such as ChatGPT (OpenAI, San Francisco, CA), Claude AI (Anthropic, San Francisco, CA), and Perplexity AI (Perplexity AI Inc., San Francisco, CA) all have the capacity to ingest diverse prompts and respond conversationally. Each of these tools can be used to constructively review author-generated content, much like a co-author might review the work of a colleague. Indeed, these tools are able to provide editorial and contextual feedback, synthesize and copyedit content, and produce suggestions of additional themes of discussion. The diversity of tasks that can be performed by these tools is considerable, but generalist chat systems can require more careful prompt engineering for application in the scientific writing space due to their training on very diverse forms of written content.

Science writing differs from other styles in the need for deep integration into the existing literature (Grogan, 2021). To support these unique needs, several science writing–specific GenAI tools exist. Tools such as Elicit AI (Elicit Research PBC, Oakland, CA), Consensus (Consensus NLP Inc., Boston, MA), and Litmaps AI (Litmaps Ltd., Wellington, New Zealand) can be used to query scientific literature, supporting users in obtaining more accessible summaries of how papers are linked together through citation networks (Litmaps), or summarizing findings across papers. In addition to querying the literature, GenAI tools have been constructed to summarize scientific content. Tools such as SciSummary (SciSummary AI, Columbus, OH), Scholarcy (Scholarcy, London, UK), and HeyScience (HeyScience SA, Lausanne, Switzerland) help distill scientific articles into more digestible synopses accessible by broader audiences. Exploration of these systems shows tremendous capacity to make science more accessible. This is true both because of the ability of these systems to translate scientific vernacular into a more readily understood phrasing, but also because of the time-saving nature of summaries. Through these attributes, these tools can appeal to a diverse audience, including those interested in faster access to content within articles, and individuals interested in more readily understandable content.

Another commonly highlighted attribute of science-focused GenAI tools is their ability to interpret findings in an unbiased manner. Despite their comfortable and conversational nature, GenAI tools are models of human discourse. As a result of their training process, they will produce content with bias, slant, or interpretation representative of the average shown in their training literature. Although they provide a fascinating mirror through which we can learn about emergent biases apparent within scientific writing, we should remember that expressing the average of biases is not the same as presenting agnostic or “bias-free” content. This is particularly the case when training data may be filtered through selection criteria that induce bias, as is likely the case with the peer-review process (i.e., publication bias).

GenAI tools focused on scientific writing are also touted as having ubiquitous understanding of scientific literature. However, different tools show variable performance in querying the scientific literature. For example, when asked to identify seminal works on GenAI in scientific article preparation, Elicit AI and Consensus only agree on one citation of the 4 or 10 citations (respectively) identified in their responses. These citations also largely differ from those highlighted by traditional search engines such as Google Scholar (https://scholar.google.com/). These differences could be driven by shifts in the training data, in the reinforcement algorithms, or in other attributes of the model training process. Without transparency in the procedures used it is challenging to have confidence that tools are derived to truly represent altruistic objectives (i.e., accessibility of science) rather than potentially biased objectives. Biased objectives could include the desire to reinforce lines of inquiry or thought for reasons other than scientific validity, as is common practice in various applications of cyberterrorism. Although GenAI tools focused on scientific literature undoubtedly make science more accessible, their value in querying and summarizing scientific content should be viewed within the limitations of their training parameters and with caution given the current limitations on information about the training and objectives of many of these systems.

Despite these nuanced limitations, it is no surprise that GenAI tools often self-identify as co-pilots. These tools represent virtual collaborators who give us their undivided attention, in real-time, whenever and however we need it. This partnership is highly valuable. However, as aptly noted in an authorless editorial (Nature, 2024) in *Nature Reviews Physics*, “AI is no substitute for having something to say.” Learning appropriate ways to use this resource to improve writing while maintaining our creativity, individuality, and unique ideas is essential. GenAI tools will, by design, produce content representative of the average human communication. Although this has value, I know few scientists who are satisfied with average.

Although specific processes often differ by laboratory and subject matter, journal article preparation typically involves the generation of tables and figures, consolidation of data into key findings, preparation of draft text sections, editing and fine-tuning text, and submitting the article for review. GenAI may help support authors in each of these phases of article preparation. The specific roles that GenAI can play will undoubtedly vary with laboratory, author skillsets and preferences, and the evolution of GenAI tool capacity. The following examples reflect the use of currently available text-based GenAI tools. Although this approach can be viewed as a “standard operating procedure,” it is recommended that authors and investigators update procedures and prompts to support their specific focus, strengths and weaknesses, approach to journal article preparation, and communication style.

Figure 1 shows a summary of some prompts to ChatGPT 4.0 and associated responses for an analysis of an example experiment. The example experiment includes a 2 × 2 factorial arrangement of treatments (X1 and X2 × Z1 and Z2) used to explain variability in outcomes Y1, Y2, and Y3. The data are synthesized in R v4.3.0 (R Foundation for Statistical Computing). Y1 is designed to have independent, additive impacts from X and Z; Y2 is influenced by an interaction of X and Z; and Y3 is independent of X and Z. In the first step, we introduce a table showing the example statistical analysis of the data and ask for a summary of key findings. The GenAI tool highlights each of the important responses: Y1 is affected independently by X and Z; Y2 is influenced by an interaction of X and Z; and Y3 is independent of X and Z. We then request help outlining a discussion, and the GenAI tool produces an outline of suggested content that should be included. GenAI tools can also be used upstream and downstream of this example; however, humans are ultimately responsible for content of the scientific article. Thus, human oversight is critical (Blau et al., 2024).

**Figure 1:**
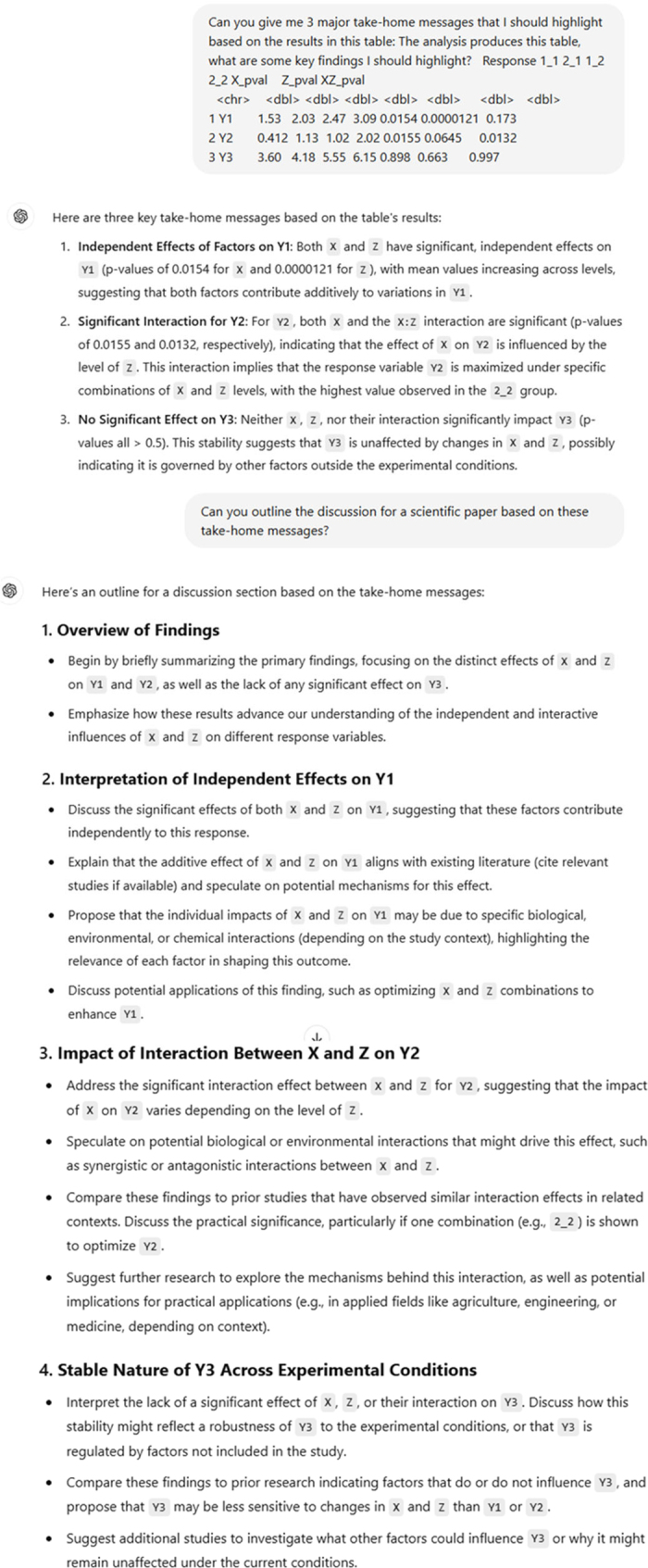
Summary of a conversation with ChatGPT 4.0 based on a request to explore the main findings in an example table of LSM and *P*-values. The tool is initially prompted to interpret the table, and then asked to provide suggestions for main points that should be highlighted in the discussion of a scientific paper. Responses show basic competency in identifying a correct interpretation of straightforward data relationships, and translating those relationships into an outline for a scientific discussion.

To support transparency and trustworthiness in the application of human oversight to GenAI-produced content, development of systematic, laboratory-specific protocols for GenAI use may be beneficial. Through such protocols, researchers can use GenAI to support efficient and streamlined development of scientific papers, while ensuring reliable human oversight. Among diverse possibilities for these protocols, text-based GenAI tools can broadly be used to synthesize text de novo or to provide feedback on text synthesized by human colleagues. Our laboratory has explored, in a limited manner, the utility of GenAI tools in these approaches. Graduate students compared outcomes from GenAI tools based on 2 protocols. In the first protocol, students created a draft of a discussion section of a scientific article and then used AI tools to edit that draft. In the second protocol, students used GenAI to generate a first draft, and they subsequently edited it manually. Students identified a number of practical limitations of contemporary GenAI in crafting scientific discussions and preferred to use the protocol where GenAI served an editorial function. In addition to the widely recognized challenges with hallucinations (i.e., generation of erroneous content) and incorrect or missing citations, students identified that GenAI tools made only limited use of the available literature. Tools missed important or seminal citations, and citations were somewhat random and sparse in GenAI-created discussion paragraphs. Additionally, GenAI tools made limited use of discipline-specific technical terms and abbreviations, were overly reliant on subsections and bulleted formats, and sacrificed detail when asked to be concise. Careful prompt engineering can help to abate some of these challenges, and further development of science writing GenAI tools will minimize the severity of these limitations. Despite these limitations, students identified that tools were very helpful in supporting logical flow and coherence, improving grammar, making linkages between thoughts, and retaining writing styles when given an example of ideal.

As of summer 2024, publisher recommendations for the use of GenAI in article preparation focused on use of GenAI to support readability and more efficient use of time. Publishers caution that GenAI should be used with human oversight and should not be used for figure generation or in the peer-review process. The process our group has adopted for use is summarized in Figure 2. Due to the individual nature of communication, the ideal use of GenAI to support individual laboratories and researchers will vary considerably. Despite variation in specific protocols preferred by individuals, the ultimate goal of a GenAI use protocol should be to ensure adequate and consistent human verification of GenAI-produced content.

**Figure 2:**
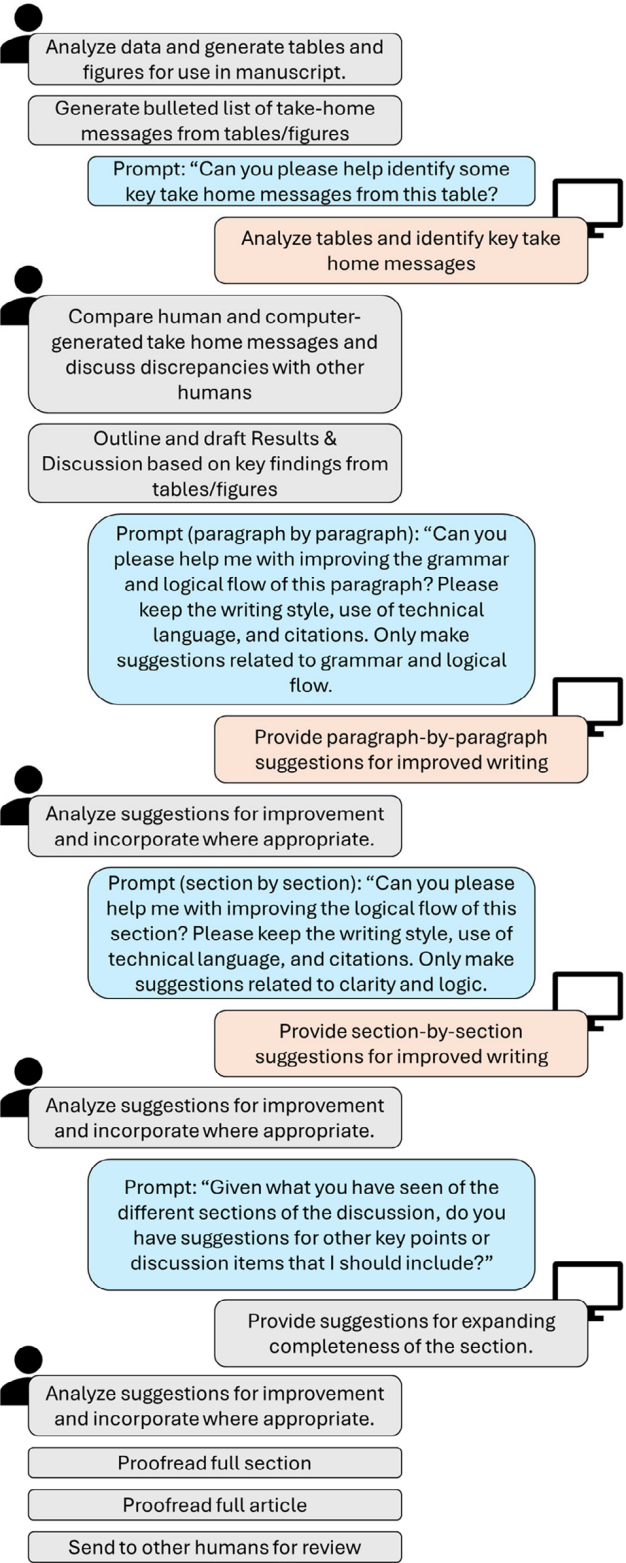
Example flow of work for human and GenAI collaboration in developing sections of a scientific article. Although the Results and Discussion section is used as an example, the same general workflow can also be applied for generating content for Introductions and Materials and Methods sections. Gray boxes reflect human tasks; blue boxes reflect example prompts to the GenAI system; and the GenAI tasks are shown in orange boxes.

Basic ethical questions on the use of GenAI in scientific article preparation have been reviewed (Al-kfairy et al., 2024; Blau et al., 2024; Mollaki, 2024). Beyond these common ethical questions, there are a number of broader considerations that pique the interest of this author. These considerations stem from the unique role that scientific articles play in our knowledge generation enterprise. Virtually since their conception, scientific articles have been held to a higher standard than other forms of human communication because they underpin the construct that we call knowledge (Farrell et al., 2017). Therefore, it seems appropriate that the use of GenAI to synthesize content for these cornerstone communications must also be held to a higher standard of scrutiny.

GenAI is a tool that holds promise to support repartitioning of time and energy resources to tasks other than generating and editing text-based communication. It is a unique attribute of our species that we focus on the development of such tools to support repartitioning of time budgets to enable us to prioritize preferred tasks (Dunbar et al., 2014). There is evidence that language and tool-making co-evolved for survival (Morgan et al., 2015), and this co-evolution was associated with the need to transfer information among individuals (Henno, 2013). Although language likely originated through verbal communication, written communication emerged as a way to support learning over space and time because it provided a means of durable knowledge transfer. Knowledge is almost by definition one of the critical evolutionary adaptations enabling species survival, and knowledge and communication are tightly linked in human societies. Indeed, in surveys of employers today, communication skills are routinely highlighted as skills essential for employment (Adams, 2014). Given the role of communication in modern society, as a prerequisite for knowledge transfer, and as a component of our evolution, increasing reliance on GenAI tools for communication may have unintended consequences. Although reliance of GenAI tools may provide opportunities for more equity among individuals with different natural competency or comfort in communication, there may also be lasting long-term impacts of overdependence on GenAI tools. Increasingly GenAI tools are being restricted behind paywalls and are the property of corporations. What are the socioeconomic implications of restricting access to these communication tools to only those with disposable income? How can the revenue-generating value of GenAI to corporations be balanced with their value to societies? These socioeconomic challenges highlight concerns related to equity and accessibility of GenAI tools and their potential for improving the human condition.

The linkages between communication, knowledge, and truth are particularly exemplified in the study of communication tools. The printing press, the first opportunity to enable mass written communication, is credited with driving new rules for how the accuracy of information was judged and provides an example of how a communications technology shifted the standard for “truth” (Farrell et al., 2017). In a more contemporary example, scholars widely agree that the expansion of digital communication technologies has changed the nature of information irreversibly (Neuman, 2016). The trends demonstrated by the Idleman Trust Barometer over the past several years suggest this shift in communication has had negative impacts on people's trust in information, and that rapid innovation risks exacerbating these trust issues, contributing to societal instability and political polarization (Edelman, 2024). Are the benefits of GenAI technologies worth these potential risks? What strategies can we rely on to mitigate the negative consequences associated with more broad-spectrum adoption of this rapidly evolving innovation? These will be critical questions over the next several years that should have unique answers specific to the use of GenAI in scientific article preparation.

This is not the first trust in information crisis experienced by humanity, nor is it likely to be the last. Similar concerns about trust in information were raised as the internet was leveraged more comprehensively as a communication platform for information sharing. To build new standards for “truth” in the age of digital communications, we formalized classifications for the type and source of information (i.e., primary sources, secondary sources, and so on). However, GenAI tools are largely built on sources scraped from the internet, which includes many more secondary and tertiary sources than primary sources. The complexity of GenAI tools is such that they do not explicitly identify which sources specifically are leveraged when generating a particular response. Considering this lack of transparency, what might be the unintended consequences of uplifting a communication technology built primarily on secondary and tertiary sources to generate primary sources (i.e., scientific journal articles)? The risks associated with this misbalance in training set information may be partially addressed through development of GenAI tools specific for scientific communication. However, the question remains as to whether scientific communications leveraging narrow AI will be able to push the standard of knowledge sufficiently to meaningfully advance our understanding of phenomena. If they are trained exclusively on existing communications, will GenAI tools be able to advance our knowledge or propose new ideas? Therein lies the transition to general, and eventually superintelligent AI.

At the root of these ramblings on communication, knowledge, and fact is the assumption that humans are truly a gold standard for science communications and codification of knowledge within scientific literature. The scholarly literature on scientific articles highlights several examples that raise concern about this assumption: publication biases, reproducibility concerns, conflicts of interest, and so on (Hesselmann et al., 2017). Undoubtedly, humans are imperfect. Humans are also resistant to change. Despite marked advancements in knowledge, analytical strategies, and computational capacity, scientists still largely rely on a scientific method underpinned by principles advocated by Aristotle and Plato (Laudan, 1968) and leverage a method of sharing science communications that was originally enacted in the 1700s (Farrell et al., 2017). The majority of the modern enterprise of science is built around the functional unit of a peer-reviewed publication (Mahapatra and Sahoo, 2023). Generation of these publications is essential to secure research funding, to show outcomes of research investments, to demonstrate programmatic productivity, and to secure long-term employment opportunities. However, the individual impact of these publications appears to be declining with time (Park et al., 2023), perhaps due to emphasis on quantity. Although likely outside the scope of this article's discussion on GenAI, and reviewed elsewhere (Suls and Martin, 2009; Kovanis et al., 2017), the advancement of GenAI tools to support scientific communications opens the door to question whether this functional unit of science (i.e., the peer-reviewed paper) is still the best our community of thinkers has to offer. In this way, perhaps GenAI will be a tool that supports the advancement of scientific communication toward a higher standard. Specifically, GenAI may help us free up intellectual power so that we can focus more intentionally on updating science communication strategies to respond to challenges such as publication bias, reproducibility, conflicts of interest, and related limitations highlighted within the existing peer-review system. Among the diverse possibilities for how this ideal might be operationalized, we could imagine the following: (1) adjusting the functional unit of science communication to annotated datasets, allowing human and GenAI teams to explore those data and support identification of insights; (2) producing meta-analytical GenAI tools to query across the scientific literature and highlight areas of consistency or inconsistency for human oversight; or (3) using tools to support the translation of scientific data into real-world impact.

As we look to the future of GenAI, there is considerable push for tools to serve as a fully automated, open-ended framework for AI-driven scientific discovery. Although this might sound like science fiction, examples and proposed approaches have already hit preprint servers (Gao et al., 2024; Lu et al., 2024; Tu et al., 2024, Tu et al., 2024). The potential ethical and practical challenges with GenAI-driven innovation are an expansion on the concept of GenAI-generated data and merit their own review. However, as a final thought relevant to this work, it is useful to point out that when the preprint publication describing GPT-3 was posted in May of 2020, some of the first commentary focused on the uncomfortable conclusion that the tool is capable of producing content that is indistinguishable from a human. To extend that conclusion to a simple statement, GenAI pushes us to question: what really makes us special? It seems to this author that continuing to seek answers to this question can only serve to make us better scientists, whether we use the tool to drive communication and innovation, or not.
